# Synthesis of Nitrogen and Sulfur Co-doped Carbon Dots from Garlic for Selective Detection of Fe^3+^

**DOI:** 10.1186/s11671-016-1326-8

**Published:** 2016-02-29

**Authors:** Chun Sun, Yu Zhang, Peng Wang, Yue Yang, Yu Wang, Jian Xu, Yiding Wang, William W. Yu

**Affiliations:** State Key Laboratory on Integrated Optoelectronics, and College of Electronic Science and Engineering, Jilin University, Changchun, 130012 China; Department of Physical Chemistry, Regional Centre of Advanced Technologies and Materials, Faculty of Science, Palacký University in Olomouc, Šlechtitelů 27, 783 71 Olomouc, Czech Republic; State Key Laboratory of Superhard Materials, and College of Physics, Jilin University, Changchun, 130012 China; Department of Engineering Science and Mechanics, The Pennsylvania State University, University Park, Pennsylvania, 16802 USA

**Keywords:** Carbon dots, Garlic, Ion detection

## Abstract

Garlic was used as a green source to synthesize carbon dots (CDs) with a systematic study of the optical and structure properties. Ethylenediamine was added into the synthesis to improve the photoluminescence quantum yield (PL QY) of the CDs. Detailed structural and composition studies demonstrated that the content of N and the formation of C–N and C=N were critical to improve the PL QY. The as-synthesized CDs exhibited excellent stability in a wide pH range and high NaCl concentrations, rendering them applicable in complicated and harsh conditions. Quenching the fluorescence of the CDs in the presence of Fe^3+^ ion made these CDs a luminescent probe for selective detection of Fe^3+^ ion.

**Electronic supplementary material:**

The online version of this article (doi:10.1186/s11671-016-1326-8) contains supplementary material, which is available to authorized users.

## Background

Fluorescent materials have drawn considerable attention owing to their potential applications in a variety of fields, such as bioimaging, optoelectronic devices, and sensing [1–7]. Semiconductor quantum dots (QDs) have become one of the most promising nanomaterials due to their high photostability, large molar extinction coefficients [8, 9], high photoluminescence quantum yields (PL QYs), and size-tunable emission [10]. However, these popular QDs have raised concerns over serious toxicity and environmental hazard [11]. In addition, their superior optical features are usually applied in organic solvents, thus restricting their direct analytical and biological applications to a great degree. Various methods have been applied to make these luminescent QDs water-soluble, such as surface passivation with hydrophilic protective layers [12–14] and water-phase synthesis. However, these protocols are compromised at the expense of reducing the PL QYs and the time-consuming, complicated, expensive processes [15].

Carbon nanoparticles, or carbon dots (CDs) are free of toxic elements. They have recently emerged as the most attractive candidates to replace QDs with outstanding advantages, such as the excellent water solubility, strong luminescence, high biocompatibility, and good photostability [16–23].

Typically, the strategies for synthesizing CDs can be divided into two major categories: top-down and bottom-up methods. Top-down methods consist of laser ablation [24], arc discharge [25], and chemical oxidation [26], where the CDs are formed by cutting a large carbon structure into small pieces. In bottom-up methods, the CDs are synthesized by carbonization of organic molecular precursors through solvothermal methods [27, 28], microwave treatment [29], ultrasonic-assisted synthetic methods [30], and so on. Among them, the top-down methods often require sophisticated and expensive energy-consuming equipment [31]. The ultrasonic-assisted synthetic methods rely on strong acids or bases [30]. Typically, CDs can be synthesized within minutes by microwave irradiation, while they suffer from uncontrollable reaction conditions. The hydrothermal route is mostly preferred because of its simplicity, controlled reaction conditions, and cost-effectiveness [31].

Despite several advancements in the field of CDs synthesis [27, 28, 32], there are some by-products using conventional chemicals, which do not meet the standard of non-toxicity. Besides, expensive precursors and complicated post-treatment processes for surface passivation also hinder the broad application of CDs. Therefore, many efforts have been made for the preparation of CDs using easily accessible and natural precursors as starting materials. For example, milk [33], potato [34], grape juice [35], lime juice [36], orange juice [37], pomelo peel [38], grass [39], willow bark [40], and even waste biomass [41] have been used as the green sources for CDs. Nevertheless, it still remains a major challenge to achieve biomass-derived CDs with high PL QYs.

Recently, heteroatom-doped CDs have been reported with enhanced optical and electronic properties. It has revealed that heteroatom doping plays a vital role in tuning compositions and structures of CDs [42, 43]. Particularly, there were intensive investigations of nitrogen-doped CDs [44], while nitrogen and sulfur co-doped CDs were rarely reported. Garlic is a cheap, easily available natural condiment, containing carbohydrate, proteins, and thiamine, and abundant in carbon, nitrogen, and sulfur elements. Consequently, dehydration, polymerization, carbonization, and passivation may involve in the formation of CDs under high temperature and pressure during the hydrothermal treatment [22, 23]. We report herein a green synthetic method for N and S co-doped CDs from garlic by a one-step hydrothermal synthesis. Systematic study of the optical and structure properties of CDs is presented in the work. In comparison to CDs prepared by other natural materials such as grass, potato, pomelo peel, willow bark, and waste biomass which have relatively low content of N and S elements, the QY of our CDs exhibited nearly double increase. The as-synthesized CDs can be applied in sophisticated and harsh conditions because of their excellent stability in a wide range of pH values and high ion strength solutions. Selective fluorescence quenching of CDs qualifies them as a probe to detect Fe^3+^ ion.

## Methods

### Materials

Fresh garlic were purchased from local supermarket (Changchun, Jilin Province). Ethylenediamine (EA) was attained from Aladdin. Na_2_S·9H_2_O were purchased from Xi Long Chemical Reagent Co., Ltd. Na_2_SO_4_, NaCl, KCl, AlCl_3_, ZnCl_2_, Ba(NO_3_)_2_, FeCl_3_, MgCl_2_, Ni(NO_3_)_2_·6H_2_O, MnCl_2_·4H_2_O, CuCl_2_·2H_2_O,PbCl_2_, and HgCl_2_ were obtained from Sinopharm Chemical Reagent Co., Ltd. and used without further purification.

### Preparation of CDs

The garlic were peeled and washed and cut into small pieces and set in the oven at 50 °C for 24 h. After dehydration, the garlic pieces were ground into fine powder in a mortar; 0.5 g of garlic powder and 10 mL of deionized water were added in an autoclave with a polytetrafluoroethylene (PTFE) inner chamber and heated at 200 °C for 6 h (for N- and S-rich CDs, additional EA, Na_2_S·9H_2_O and Na_2_SO_4_ were added into the reaction). After the reaction, the autoclave was cooled to room temperature naturally. The reaction product was centrifuged at 5000 rpm for 10 min to remove the black precipitates, and then, the resulted supernatant was purified by filtering out large-sized carbon nanoparticles using a syringe filter with pores of 0.22 μm. Finally, the product was subjected to dialysis (MWCO = 1000 Da) in order to obtain the pure CDs.

### Characterization

Fluorescence emission spectral measurements were carried out using a Shimadzu RF-5301 fluorescence spectrophotometer. Absorbance spectra were acquired by using a Shimadzu UV-3600 spectrophotometer. The surface morphology of the as-prepared CDs was investigated using a transmission electron microscope (TEM) (JEOL). X-ray diffraction (XRD) patterns of CDs were obtained using a Bruker D8 Advance X, Pert diffractometer (Cu Kα: *λ* = 1.5406 Å), in the range of 10°–70° at a scan rate of 4°min^−1^. Atomic force microscopy (AFM) images were recorded with a Veeco DI-3100 instrument. Fourier transform infrared spectroscopy (FTIR) was performed on an FTIR spectrophotometer (IFS-66 V/S). X-ray photoelectron spectroscopy (XPS) was conducted on an ESCALAB250 spectrometer. Binding energy of C 1 s at 284.7 eV was set as the calibration. The absolute PL QYs of the solution sample were measured by a fluorescence spectrometer (FLS920P, Edinburgh Instruments) equipped with an integrating sphere with its inner face coated with BENFLEC.

### Metal Ion Detection of the CDs

Many different kinds of metal cations have been applied for the detection, such as Na^+^, K^+^, Al^3+^, Zn^2+^, Ba^2+^, Fe^3+^, Mg^2+^, Ni^2+^, Mn^2+^, Cu^2+^, Pb^2+^, and Hg^2+^. Briefly, a metal salt aqueous solution (250 μM, 2 mL) was mixed with a CD solution (0.05 mg/mL, 2 mL). After the mixture was vibrated for 5 min, the fluorescence spectra of the mixture were recorded.

### pH Stability of CDs

HCl (2 M) or NaOH (2 M) was used to adjust the pH of the resultant CD solution (0.05 mg/mL, 2 mL), and the mixed solution was vibrated for 5 min. Then, the fluorescence spectra of the mixture were carried out.

### Ion Strength Stability of CDs

NaCl aqueous solution (2 mL) with certain concentrations was mixed with a solution of CDs (0.05 mg/mL, 2 mL). Then, the fluorescence spectra of the mixture were recorded after the mixed solution was equilibrated for 5 min with vibrating.

## Results and Discussion

**Fig. 1:**
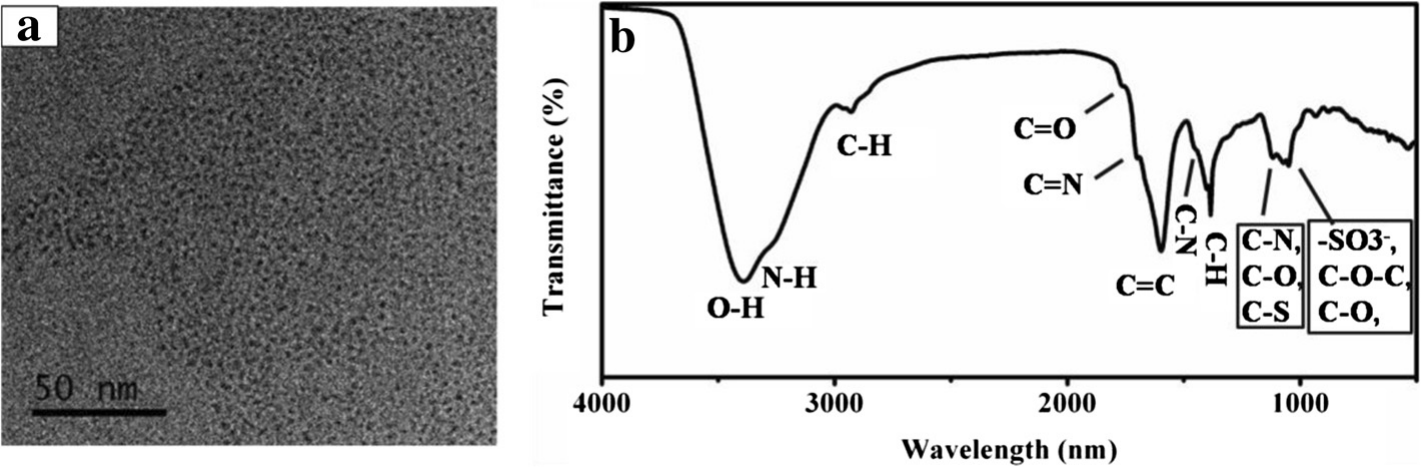
TEM image and FT-IR spectrum of CDs. **a** TEM image of the CDs. **b** FT-IR spectrum of CDs

Garlic is a natural world-wide edible condiment which is abundant in carbon, sulfur, and nitrogen elements and thus was used here as carbon source to synthesize the N- and S-doped CDs by a one-step hydrothermal treatment. After hydrothermal reaction, a light brown aqueous solution was produced indicating successful carbonization of the garlic. As shown in Fig. 1a, the CDs had roughly spherical shapes and were well dispersed with diameters in the range of 1–3 nm, which was supported by AFM results (Additional file 1: Figure S1a, c). Two broad peaks at 2*θ* of 25° and 44°can be seen from their XRD pattern (Additional file 1: Figure S1b), which is similar to other reported CDs [45]. In accordance with other reported CDs [39], the QY of CDs was enhanced from 5.1 to 10.5 %, while the emission wavelength and FWHM (full width at half maximum) were nearly the same with the increasing reaction temperature from 150 to 200 °C (Additional file 1: Table S1). The QYs of CDs prepared from different natural materials were shown in Additional file 1: Table S2. As can be seen, the QY of CDs doped with nitrogen and sulfur is better than grass, potato, pomelo peel, willow bark, and waste biomass which have relatively low content of nitrogen and sulfur.

The structure and components of CDs were identified by FTIR and XPS. As illustrated in Fig. 1b, the broad band at 3399~3283 cm^−1^ was attributed to O-H and N-H bonds [46]. A small band at 2931 cm^−1^ was ascribed to the C-H bonds [44]. The peak at 1769 cm^−1^ could be ascribed to C=O stretching vibration, while the peak at 1608 cm^−1^ was attributed to the C=C [27, 42]. Moreover, two bands at 1704 and 1454 cm^−1^ were assigned to the C=N and C–N stretching vibrations, respectively [42, 47]. In addition, the peak at 1121 cm^−1^ could be identified as the C–O, C–N, and C–S bonds [48] and the peak at 1048 cm^−1^ could be ascribed to –SO^3−^, C–O–C, and C–O bonds [44, 49].

**Fig. 2:**
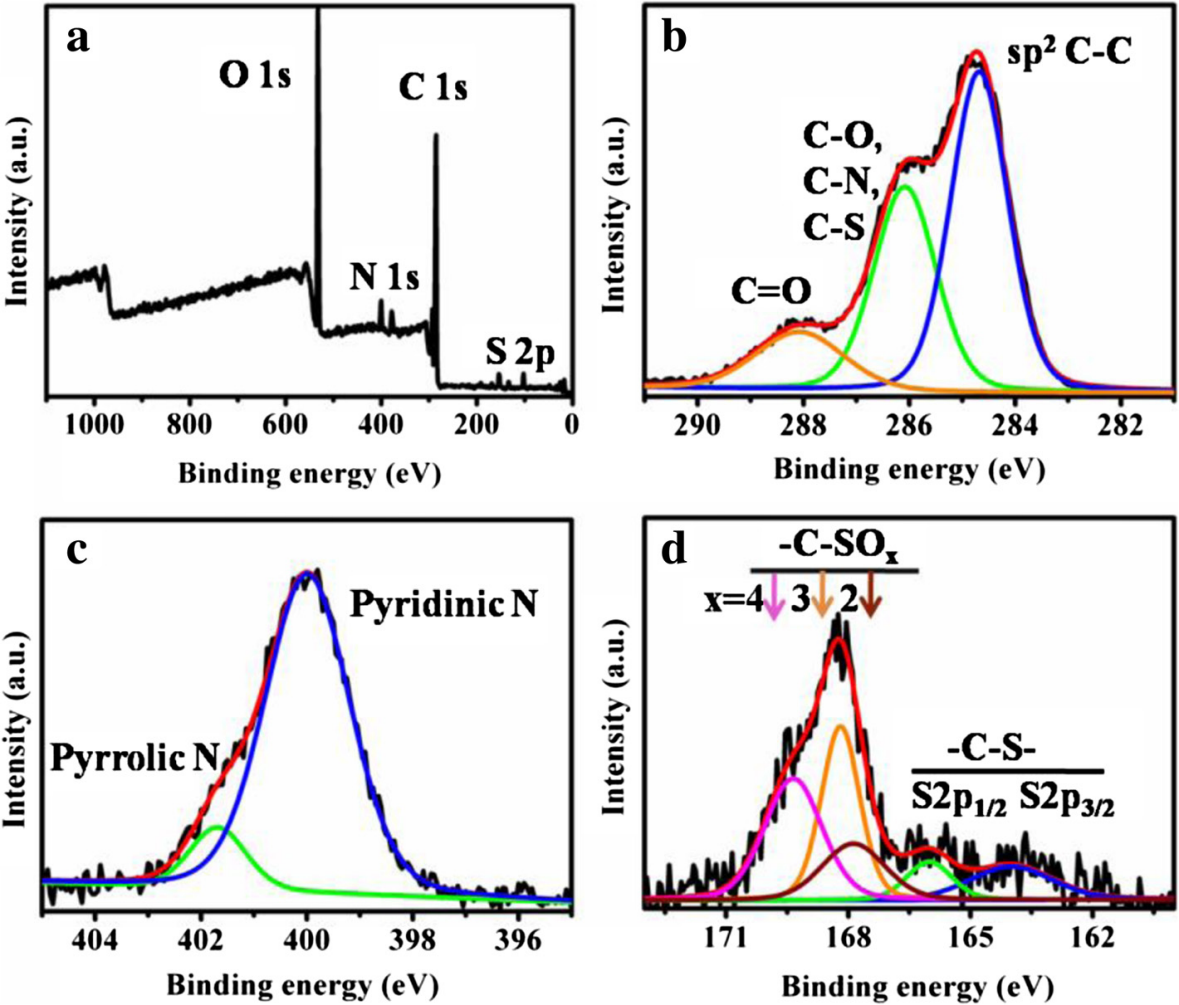
XPS spectra of CDs. XPS spectra of CDs (**a**) and high-resolution spectra of C1s (**b**), N1s (**c**), and S 2p (**d**)

As Fig. 2a shows, four peaks with the binding energies of 532.0, 399.9, 284.8, and 168.3 eV can be observed, representing the presence of O 1s, N 1s, C 1s and S 2p, respectively. The high-resolution C1s spectrum in Fig. 2b has three peaks at 284.6, 286, and 288 eV, which were attributed to graphitic structure (sp^2^ C=C), C–S, C–N, C–O (epoxy and alkoxy), and C=O species, respectively [27]. The N1s spectrum exhibited two main peaks (Fig. 2c), revealing the presence of both pyridinic N (400 eV) and pyrrolic N (401.7 eV) [27, 44], which was consistent with the FTIR results. According to Fig. 2d, the S 2p spectrum mainly consisted of three peaks centered at 164, 166, and 168.2 eV. The former two peaks could be attributed to 2p_3/2_ and 2p_1/2_ of the –C–S– covalent bond because of their spin-orbit couplings [44]. The latter peak could be deconvoluted into three components at 167.8, 168.1, and 169.3 eV, representing to the –C–SO_*x*_– (*x* = 2, 3, 4) species [44].

**Fig. 3:**
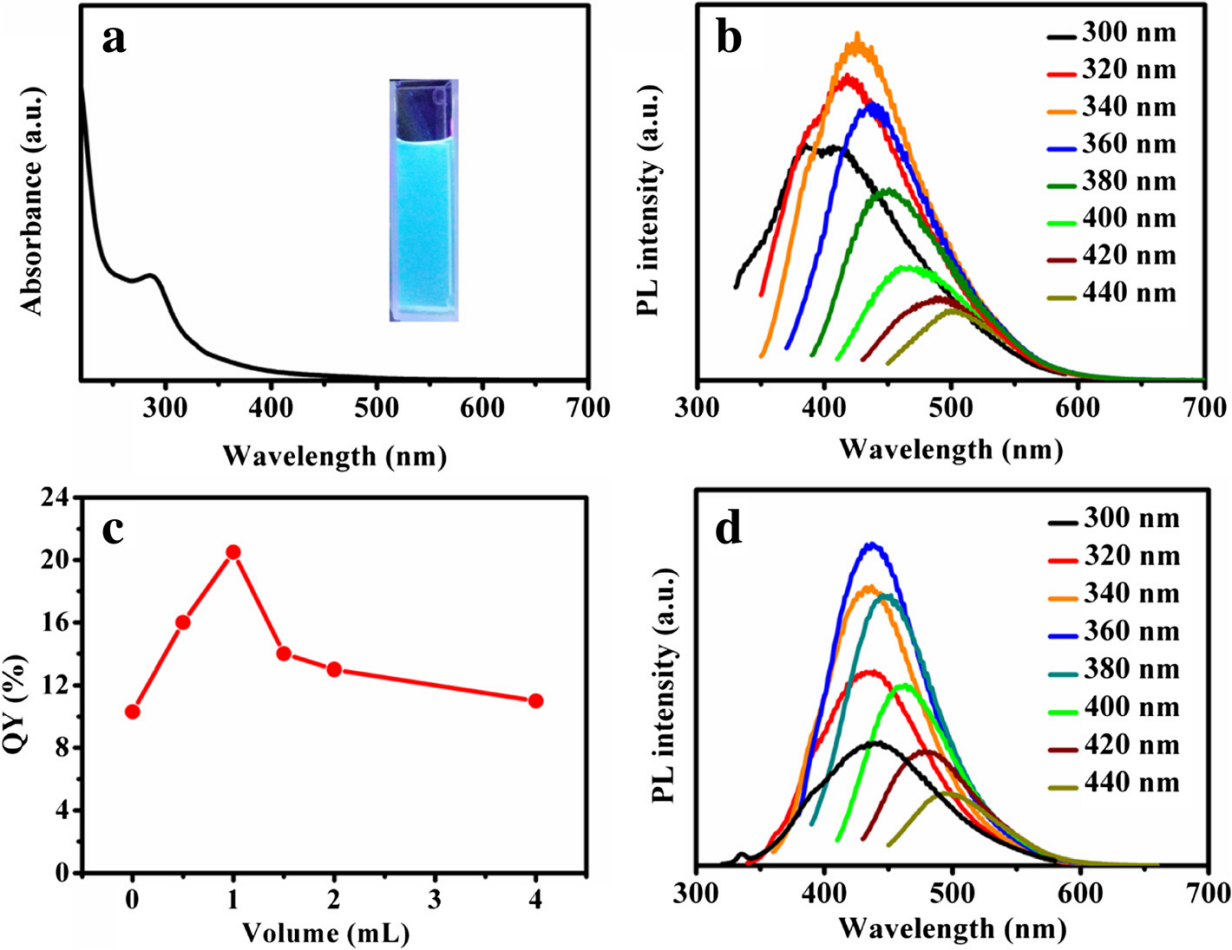
Photoluminescence properties of CDs. **a** UV-vis absorption of CDs (*inset* was the photo under UV light). **b** PL emission spectra of CDs under different excitation wavelength from 300 to 440 nm. **c** PL QY of CDs prepared by adding different volumes of EA. **d** PL emission spectra of CDs with 1 mL EA added (CD-1) under different excitation wavelength from 300 to 440 nm

To further explore the optical properties of the CDs, absorption and photoluminescent (PL) spectra were studied as shown in Fig. 3. Absorption peak centered at 287 nm could be attributed to the presence of carbonyl or conjugated carbonyl groups. For the PL spectra, the optimal emission wavelength of CDs was centered at 426 nm with the excitation of 340 nm, showing the excitation wavelength dependent emission, which is similar to other reported CDs (Fig. 3b) [31, 42, 44, 50]. The dual emissive peaks (385 and 409 nm) can be seen from the PL spectrum (black line) with the excitation of 300 nm. The emission peaked at 385 nm gradually disappears, with the excitation wavelength changing from 320 to 440 nm. According to the previous studies [21, 51], the emission peak can be divided/fitted into several individual peaks with different energies. Under the short wavelength excitation, the excitation energy is high enough to excite all the several individual peaks. However, with the increase of the excitation wavelength, the excitation energy is not sufficient to excite the high energy transitions. As shown in the inset of Fig. 3a, bright blue photoluminescence of CDs can be easily recognized by eyes under UV light (365 nm). The QY of the CDs in aqueous solution was 10.5 %, which is comparable to previous reports, too [31, 38, 40, 41, 50].

Heteroatom-doped CDs can modulate compositions and structures of CDs to enhance the optical and electronic properties [42, 43]. In order to elucidate the effect of N element and further increase the QY of the obtained CDs, an additional N-containing chemical was added into the hydrothermal synthesis. EA is a commonly used N precursor to synthesize N doped CDs, which was chosen in this work. In detail, different volumes of EA were added to the garlic powder and heated at 200 °C for 6 h. As shown in Fig. 3c, the QY firstly increased with the EA added and then dropped gradually with more EA. The highest QY could be tuned to 20.5 % with 1 mL of EA. When the ratio of EA was high, the CDs would be carbonized hardly [28] which led to lower QYs.

The absorption of two typical samples (1 mL EA denoted as CD-1 and 4 mL EA denoted as CD-4) was studied as shown in Additional file 1: Figure S2a. A new absorption band at 358 nm appeared for the EA addition, which is related to surface states [16, 27, 51]. Absorption and QY of CD-1 with different reaction times were shown in Additional file 1: Figures S2b and S3a. When the reaction time was 2 h, the absorption peak at 287 nm dominated and the QY was very low. However, when the reaction time was prolonged to 3 h, the absorption peak at 358 nm appeared and the QY was still not high. With the reaction time further prolonged, the absorption peaks remained unchanged. As shown in Additional file 1: Figure S3a, the QY firstly increased with the increase of reaction time up to 6 h; then, no apparent change of QY was observed with the reaction time to 8 h. Additional file 1: Figure S3b shows that the particle size of CD-1 was the same as that of the CDs without EA addition. The PL property of CD-1 was shown in Fig. 3d. The emission had a redshift of 10 nm compared to the one without EA.

The XPS and FTIR experiments were also carried out to identify the structure and components of these EA-assisted CDs. The band at 1769 cm^−1^ corresponding to C=O almost disappeared, and the bands at 1704 and 1454 cm^−1^corresponding to C=N and C–N became more obvious in the FTIR spectra of CD-1 and CD-4 (Additional file 1: Figure S4), suggesting that N atom gradually substituted O atom during the dehydrolysis process, which was consistent with the elemental compositions result (Additional file 1: Table S3). The content of pyrrole N of CD-1 was higher than that of CD-4 which could be seen from Additional file 1: Figures S5 and S6. No other states of N and distinct differences were observed in the XPS, which proved that N atoms entered CD framework through the dehydrolysis and the content of N was helpful to acquire high QY.

Based on the above results, the role of EA in the synthesis of CDs could be explained as below. Firstly, the –COOH or the C=O, C–O of the garlic may react with EA, facilitating dehydrolysis and carbonization process. Then N atoms substitute part of O atoms to form amides, which leads to a newly formed surface state induced by N atoms. In this case, surface passivation may facilitate a high yield of the radiative recombination and depress the non-radiative recombination [52]. As a consequence, the PL QY of the resulting CDs increases as the N content of CDs increases.

Compared to the role of N, S shows relatively weak influence. Firstly, Na_2_S·9H_2_O was used as the S precursor to synthesize S-doped CDs. However, as shown in Additional file 1: Figure S7a, the QY of the CDs was not enhanced; conversely, the QY of the CDs declined with the increasing mass of S precursor. This is mainly because Na_2_S is a strong base which has an effect on the PL intensity of the CDs. Then Na_2_S was substituted by Na_2_SO_4_ whose pH was neutral. As shown in Additional file 1: Figure S7b, the QY of the CDs increased slightly with the increasing mass of S precursor, which is not as effective as the N precursor. The absorption and PL spectra of two S precursors were studied as shown in Additional file 1: Figure S8. For the Na_2_S case, the absorption was the same to the one without S precursor when the amount of Na_2_S was small. However, with the increasing of Na_2_S amount, the absorption peak became less clear and changed as the absorption of CDs in pH value of 13. This is mainly because Na_2_S is a base which could change the pH value of the reaction solution and led to the decline of PL intensity. The absorption did not change with the addition of the Na_2_SO_4_, indicating no energy level changed. The PL spectra of these two S precursors were the same, and a little difference was achieved from the CDs without S. Therefore, compared to the N precursor, the S precursors have inconspicuous influence on the dehydrolysis and carbonization process.

**Fig. 4:**
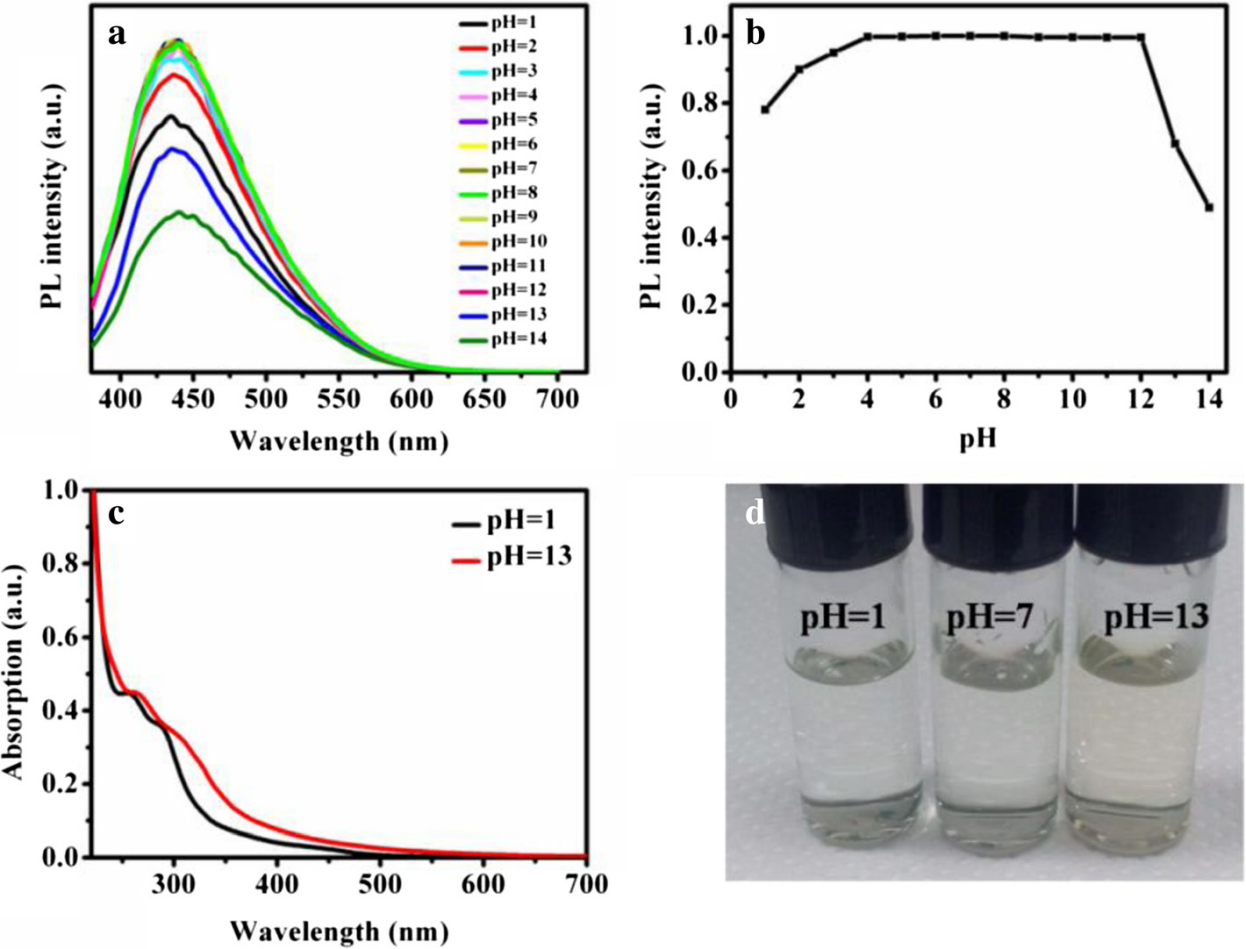
The effect of pH for the CDs. **a** PL spectra for various pH in the range 1–14. **b** The PL intensity of pH variation. **c** The absorption spectra of CDs in aqueous solution under pH = 1 and 13. **d** The photographs of CDs under room light at pH = 1, 7, and 13

The pH behavior of the CDs was explored (Fig. 4). The CDs had high stability against pH variation in the range of 3–12. As illustrated in Fig. 4c, in aqueous solution with high pH 13, the absorption peak became less clear and exhibited an obvious red-shift by comparison with the CDs of pH 1 which gave rise to the color change of CDs under daylight (Fig. 4d). The carboxyl group on the surface of CDs turned to electronegative with higher pH, which could lead to the change of the absorption peak [53].

**Fig. 5:**
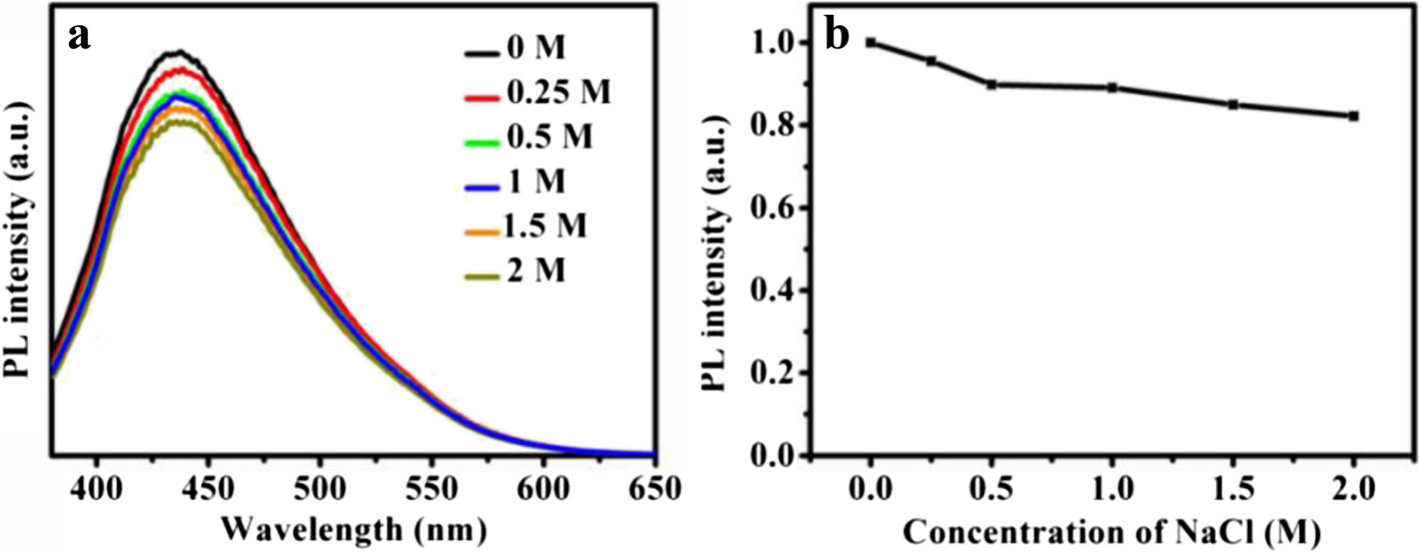
The effect of ion strength for the CDs. **a** PL spectra in NaCl solutions with different concentrations; **b** the plot of PL intensity with NaCl variation

The influence of different ionic strengths on the PL intensity of CDs was also evaluated in NaCl solution with varying concentrations from 0 to 2.0 M. As shown in Fig. 5a, there are nearly no changes in either the PL intensities or the peak characteristics, which is beneficial to use these CDs in salt solutions such as buffers. The stability of the CDs in salt and wide pH conditions ensures that they can be applied in sophisticated and harsh conditions. Additionally, no obvious photobleaching phenomenon was observed with a continuous exposure under UV excitation for 24 h, indicating excellent photostability of these CDs.

**Fig. 6:**
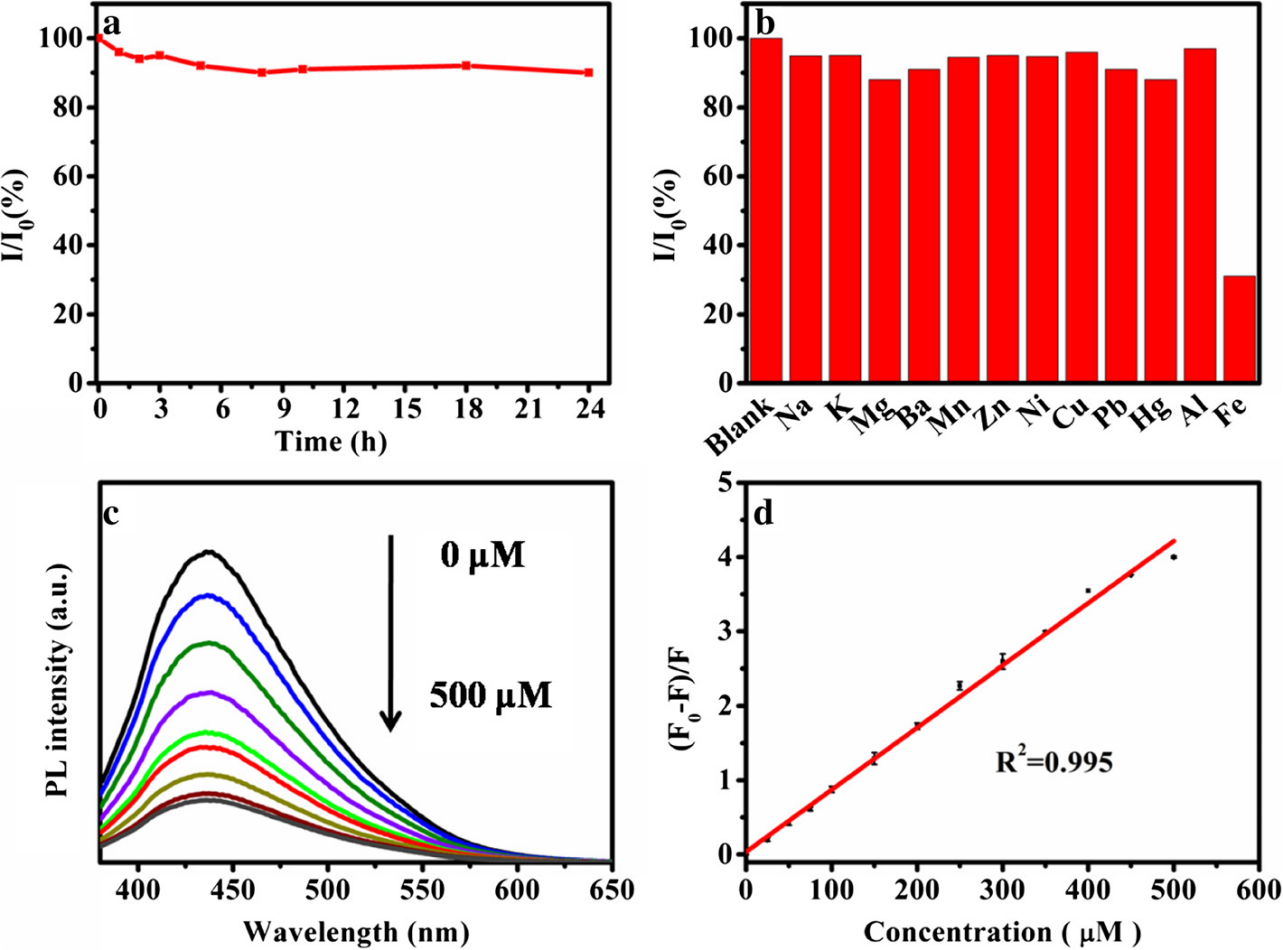
Photostability and ion detection for CDs. **a** Photostability of CDs under UV excitation at various time. **b** The metal stability of CDs in the presence of different metal ions with the concentration of 250 μM. **c** PL spectra of CDs with different Fe^3+^ concentrations (0 to 500 μM). **d** Plot of (*F*_0_–*F*)/*F* with different Fe^3+^ concentrations (0 to 500 μM)

Recently, selective detection of metal ions using fluorescence materials has been the subject of many studies [31, 38, 39, 43, 50, 54]. The selective metal detection of CDs was monitored in the presence of different metal ions as shown in Fig. 6b. Obviously, Fe^3+^ ion showed the obvious fluorescence quenching effect on CDs, while the influence of other metal ions was almost negligible because the exceptional coordination between Fe^3+^ ion and hydroxyl group of CDs [31, 43, 50]. The fluorescence quenching effect of Fe^3+^ was performed to explore the sensitivity of CDs toward Fe^3+^ ion concentration. As shown in Fig. 6c, fluorescence intensity decreased with increasing Fe^3+^ concentration. Fig. 6d further represents the relation of the relative intensity ((*F*_0_–*F*)/*F*) with different Fe^3+^ concentrations. The fluorescence quenching efficiency can be further described by the Stern–Volmer plot with a perfect linear behavior (the linear correlation coefficient was 0.995) in the range of Fe^3+^ concentrations from 0 to 500 μM. The Stern–Volmer equation was thus achieved as *F*_0_/*F* = 1.035 + 0.008 [Fe^3+^], where *F*_0_ and *F* were the fluorescence intensities of CDs in the absence and presence of Fe^3+^ and [Fe^3+^] represented the concentration of Fe^3+^. The detection limit was as low as 0.2 μM based on the calculation of 3*σ*/*m*, where *σ* was the standard deviation of blank sample signal and *m* was the slope of the linear fit. Additional file 1: Table S4 shows the comparison of Fe^3+^ detection with different CDs. As can be seen, the detecting limit of 0.2 μM compares favorably with those of previous reports for Fe^3+^ detection [31, 43, 55, 56].

## Conclusions

A direct, simple, and green synthetic approach for CDs using garlic as carbon source was presented. The content of N and the formation of C–N, C=N were critical to improve the PL QY. The as-prepared CDs showed no obvious photobleaching toward UV light. Fluorescence quench effect of the CDs in the presence of Fe^3+^ ion provided a platform for detecting Fe^3+^ ion from 0.2 to 500 μM.

## Additional file

Additional file 1: Figures S1–S8 and Tables S1–S4. Figures depicting AFM and XRD (Figure S1), absorption of different volume of EA and different reaction time (Figure S2), QY of different reaction time and TEM of CD-1 (Figure S3), FTIR of CD-1 and CD-4 (Figures S4), XPS spectra of CD-1 and CD-4 (Figures S5 and 6), QY of different amount of S source (Figures S7), absorption and PL of S source (Figures S8), different reaction temperature (Table S1), comparison of different natural materials (Table S2), elemental compositions (Table S3), comparison of Fe^3+^ detection (Table S4)(DOC 2718 kb)
