# Supplementary material for: Synthesis of Nitrogen and Sulfur Co-doped Carbon Dots from Garlic for Selective Detection of Fe3+
Source: Nanoscale Res Lett. 2016 Feb 29;11:110. doi: 10.1186/s11671-016-1326-8 (PMC4770002; doi:10.1186/s11671-016-1326-8)
Supplement: Additional file 1: — Figures S1–S8 and Tables S1–S4. Figures depicting AFM and XRD (Figure S1), absorption of different volume of EA and different reaction time (Figure S2), QY of different reaction time and TEM of CD-1 (Figure S3), FTIR of CD-1 and CD-4 (Figures S4), XPS spectra of CD-1 and CD-4 (Figures S5 and 6), QY of different amount of S source (Figures S7), absorption and PL of S source (Figures S8), different reaction temperature (Table S1), comparison of different natural materials (Table S2), elemental compositions (Table S3), comparison of Fe3+ detection (Table S4)(DOC 2718 kb) [file 11671_2016_1326_MOESM1_ESM.doc]

**Supporting Information**

**Synthesis of nitrogen and sulfur co-doped carbon dots from garlic for selective detection of Fe^3+^**

Chun Sun^1^, Yu Zhang^1^,*, Peng Wang^3^, Yue Yang^1^, Yu Wang^2^,*, Jian Xu^4^, Yiding Wang^1^ and William W. Yu^1^

1. State Key Laboratory on Integrated Optoelectronics, and College of Electronic Science and Engineering, Jilin University, Changchun 130012, China

2.Regional Centre of Advanced Technologies and Materials, Department of Physical Chemistry, Faculty of Science, Palacký University in Olomouc, Šlechtitelů 27, 783 71 Olomouc, Czech Republic.

3.State Key Laboratory of Superhard Materials, and College of Physics, Jilin University, Changchun 130012, China.

4.Department of Engineering Science and Mechanics, The Pennsylvania State University, University Park, Pennsylvania 16802, United States

* Corresponding authors. E-mails: [yuzhang@jlu.edu.cn](mailto:yuzhang@jlu.edu.cn) (Y. Zhang)

[yu.wang@upol.cz](mailto:yu.wang@upol.cz) (Y. Wang).

**
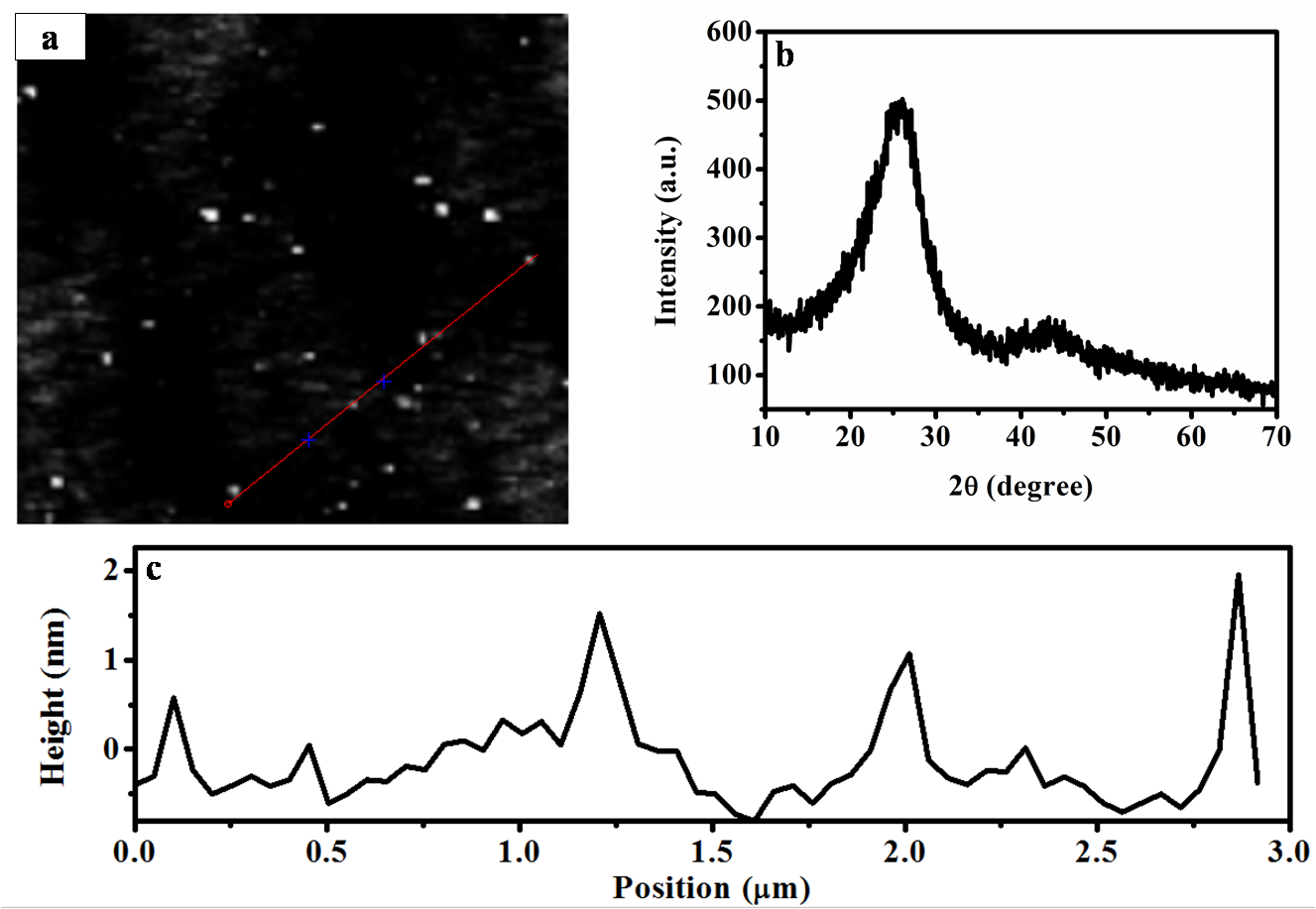
**

**Fig. S1 (a)** AFM image of CDs on Si substrates and **(c)** their height distribution; **(b)** XRD pattern of the CDs.

Table S1. Comparison of CDs prepared at different reaction temperature for 6 h

| Temperature (ºC) | QY (%) | Emission wavelength (nm) | FWHM (nm) |
| --- | --- | --- | --- |
| 150 | 5.1 | 432 | 105 |
| 180 | 9.7 | 436 | 102 |
| 200 | 10.5 | 435 | 100 |

Table S2. Comparison of different of CDs prepared from different natural materials

| Carbon source | Size (nm) | Maximum emission peak (nm) | Quantum yield (%) | Synthesis method | Ref. |
| --- | --- | --- | --- | --- | --- |
| Jinhua bergamot | 10 | 440 | 50.78 | Hydrothermal |  |
| Willow bark | 1-4 | 437 | 6 | Hydrothermal |  |
| Grass | 2 | 443 | 6.2 | Hydrothermal |  |
| Pomelo peel | 2-4 | 444 | 6.9 | Hydrothermal |  |
| Potatoe | 1-3 | 442 | 2.8 | Hydrothermal |  |
| Candle soot | 1 | 495 | 1.9 | Refluxing with HNO_3_ |  |
| Winter melon | 4.5-5.2 | 448 | 7.51 | Hydrothermal |  |
| Waste biomass | 2-4 | 400 | 2.85 | Hydrothermal |  |
| Orange juice | 2.5 | 455 | 26 | Hydrothermal |  |
| Lime juice | 7.6±2.7 | 450 | 40 | Hydrothermal |  |
| Grape juice | 2.7±0.5 | 419 | 13.5 | Hydrothermal |  |
| Milk | 3 | 454 | 12 | Hydrothermal |  |
| Coriander leaves  Garlic | 2.387  1-3 | 400  426 | 6.48  10.5 | Hydrothermal  Hydrothermal | This work |


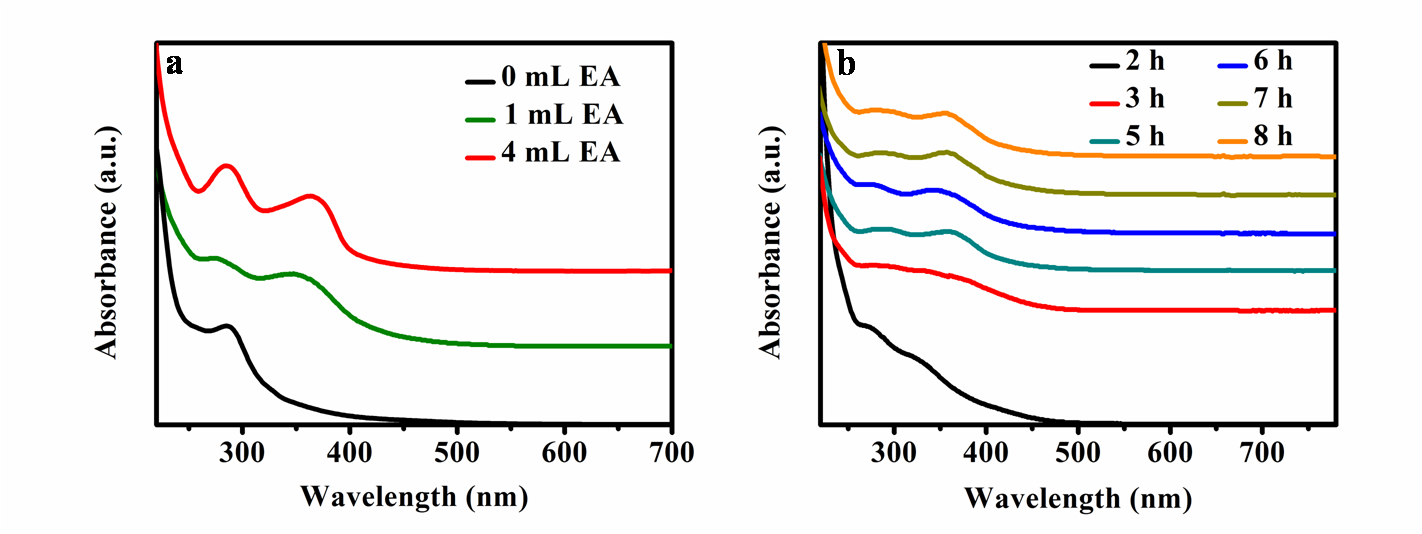


**Fig. S2 (a)** Absorption of CDs prepared by adding different volume of EA; **(b)** absorption of CD-1 prepared by different reaction time.


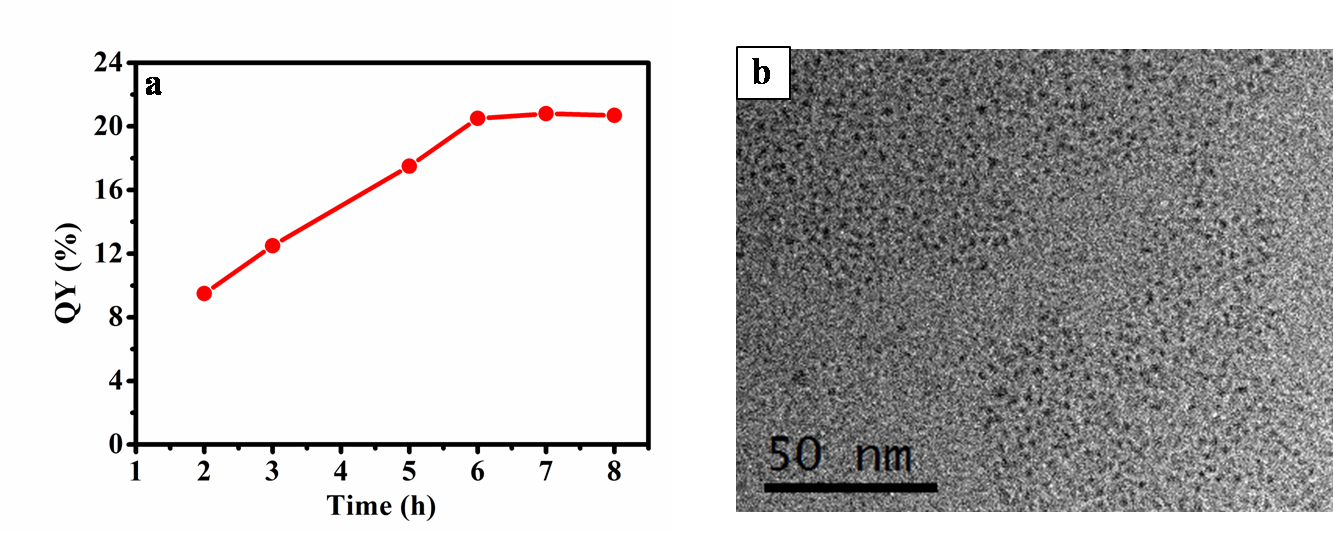


**Fig. S3 (a)** QY of CD-1 prepared by different reaction time; **(b)** TEM of CD-1 at 6 h.


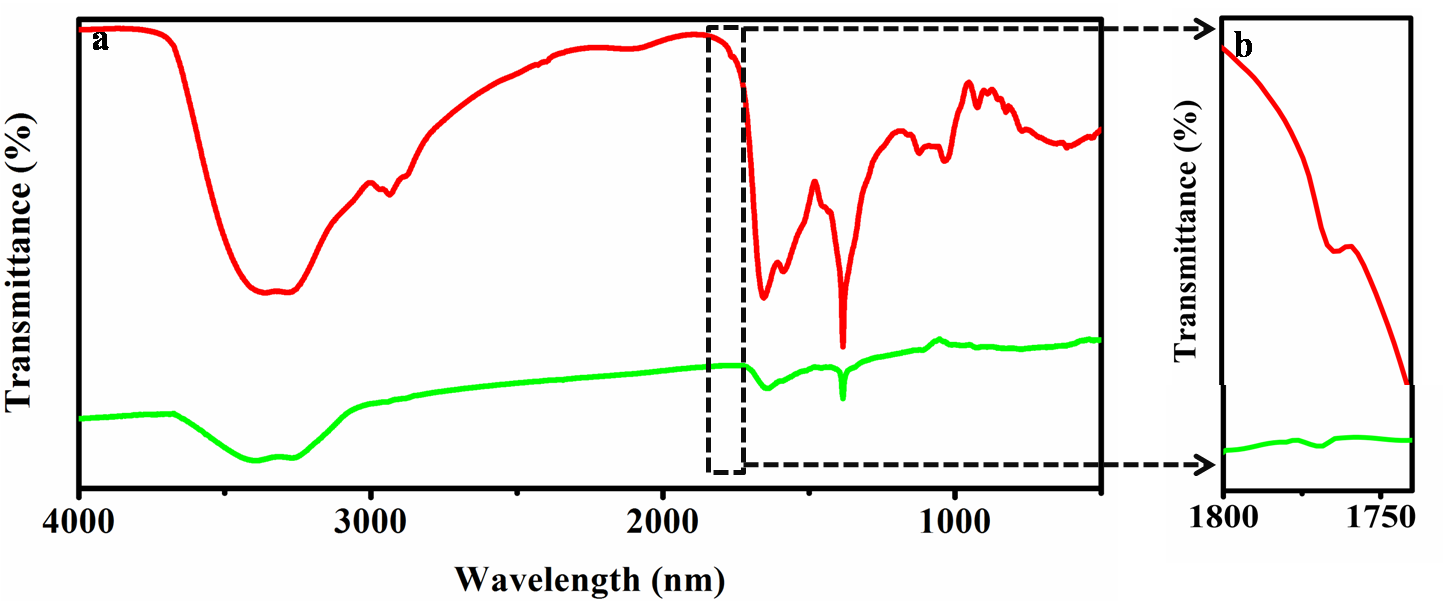


**Fig. S4** FTIR of CD-1 (red) and CD-4 (green).


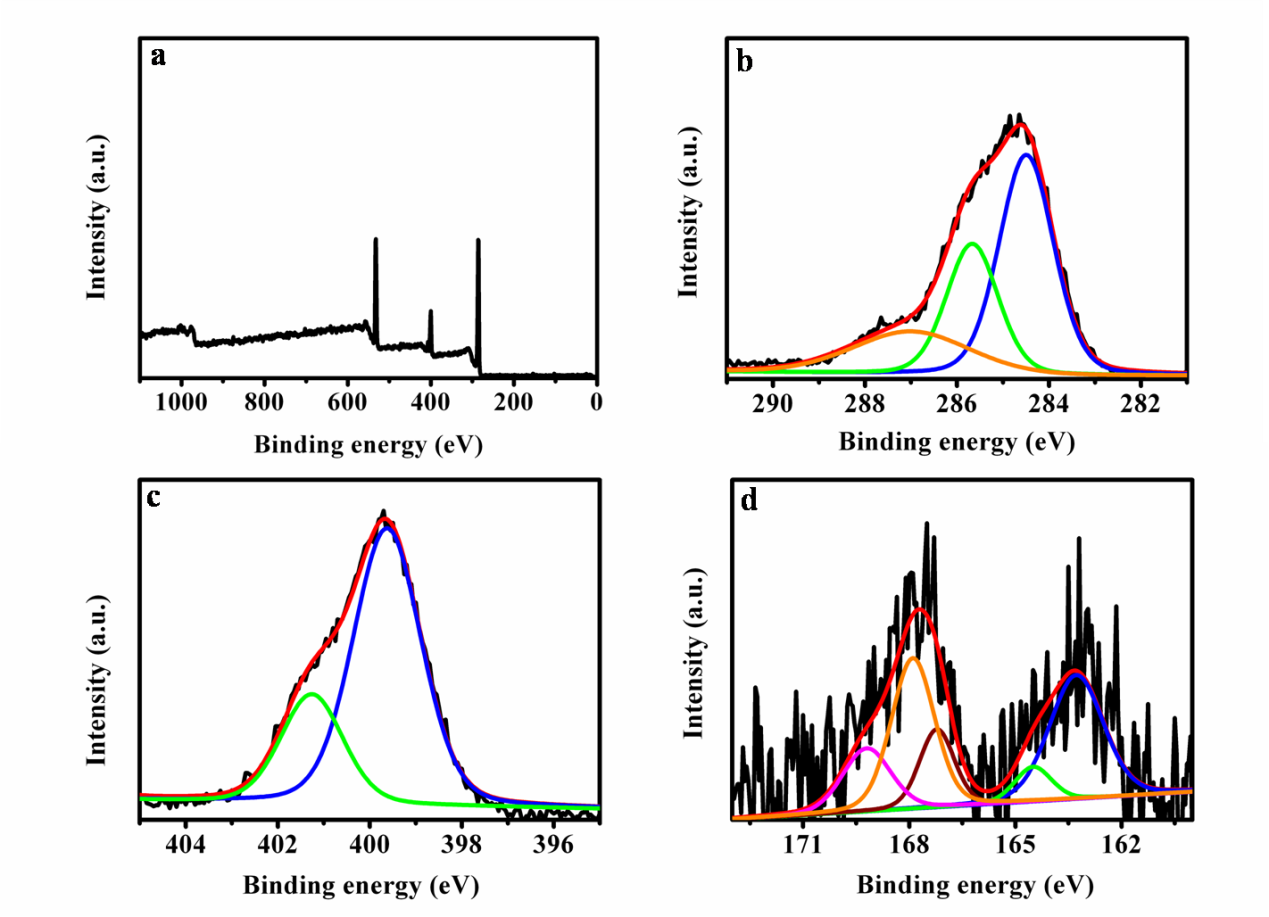


**Fig. S5** XPS spectra of CD-1 **(a)**, and the high-resolution spectra of C 1s **(b)**, N 1s **(c)** and S 2p **(d)**.


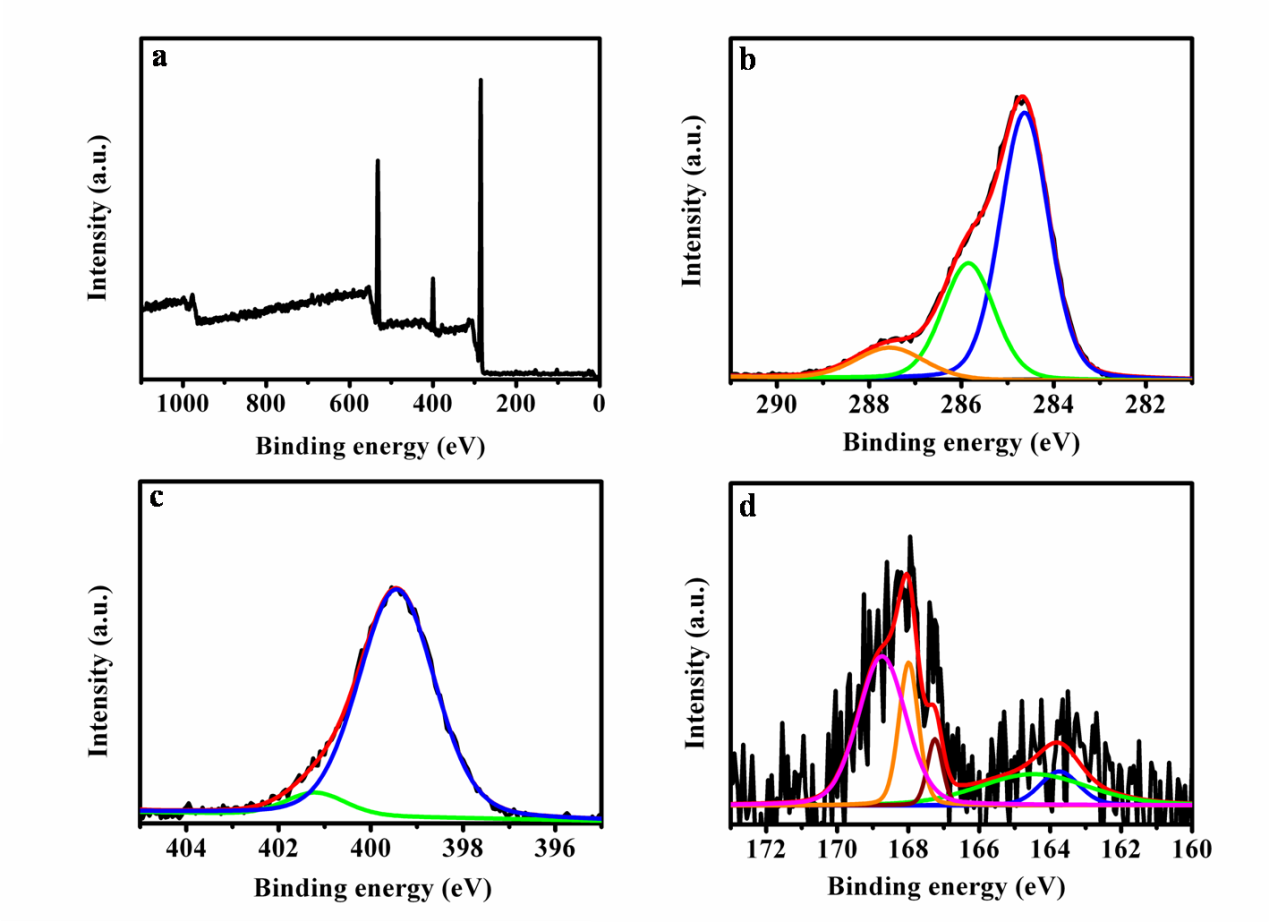


**Fig. S6** XPS spectra of CD-4 **(a)** and the high-resolution spectra of C 1s **(b)**, N 1s **(c)** and S 2p **(d)**.

Table S3. Elemental compositions of the CDs prepared with different EA volume

| Volume (mL) | C (atom%) | N (atom%) | S (atom%) |
| --- | --- | --- | --- |
| 0 | 64 | 4.61 | 0.65 |
| 1 | 64.96 | 15.15 | 0.4 |
| 4 | 74.14 | 8.55 | 0.25 |


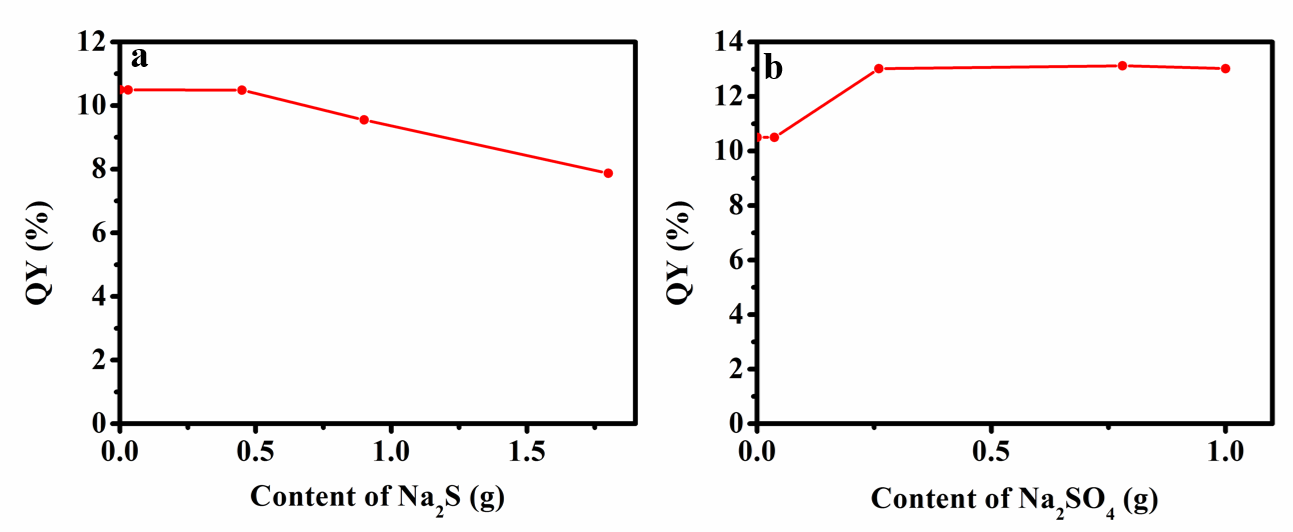


**Fig. S7 (a)** QY of CDs prepared by adding different amount of Na_2_S; **(b)** QY of CDs prepared by reacting with different amount of Na_2_SO_4_.


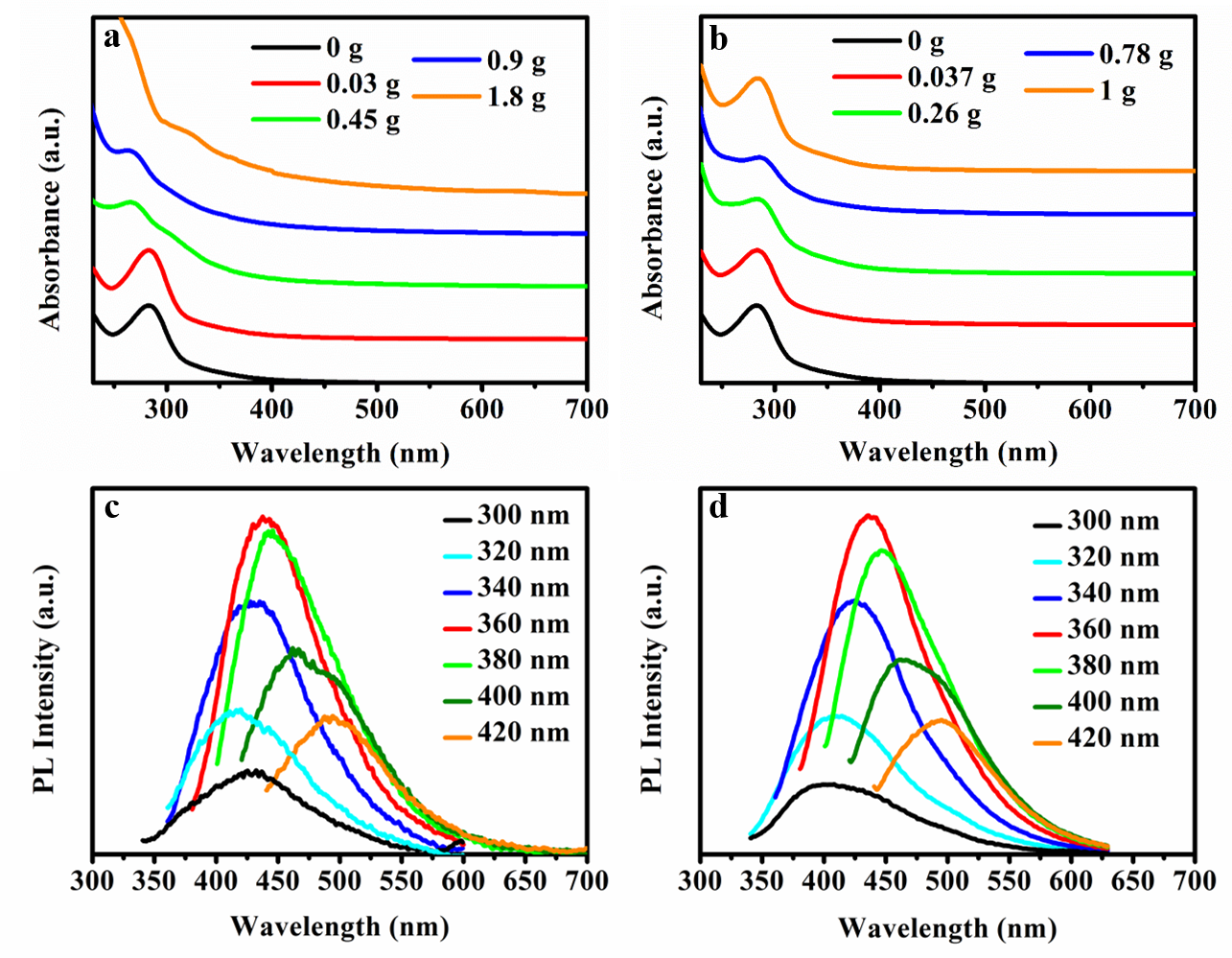


**Fig. S8 (a)** UV-Vis absorption of CDs prepared by adding different content of Na_2_S; **(b)** UV-Vis absorption of CDs prepared by reacting with different content of Na_2_SO_4_; **(c)** PL of CDs prepared by adding 0.9 g Na_2_S•9H2O; **(d)** PL of CDs prepared by reacting with 0.26 g of Na_2_SO_4_.

Table S4. Comparison of different CDs of Fe^3+^ detection

| Carbon source | Detect limit | Quantum yield (%) | Synthesis method | Ref. |
| --- | --- | --- | --- | --- |
| Citric acid and urea  Dopamine  β-Cyclodextrin | 0.04 µM  0.32 µM  0.8 µM | 14  6.4  6.4 | Microwave  Hydrothermal  Reflux with acid |  |
| Ionic liquids | 20 nM | 25.8 | Microwave |  |
| Histidine | 10 nM | 8.9 | Microwave |  |
| Folic acid | 2 ppm | 68 | Hydrothermal |  |
| Sodium citrate and sodium thiosulfate | 0.1 mM | 67 | Hydrothermal |  |
| Jinhua bergamot | 0.075µM | 50.78 | Hydrothermal |  |
| Coriander leaves  Garlic | 0.4 µM  0.2 µM | 6.48  10.5 | Hydrothermal  Hydrothermal | This work |

**References**

1. Yu J, Song N, Zhang Y-K, Zhong S-X, Wang A-J, Chen J. Green preparation of carbon dots by Jinhua bergamot for sensitive and selective fluorescent detection of Hg^2+^ and Fe^3+^. Sensors and Actuators B: Chemical. 2015;214:29-35.

2. Qin X, Lu W, Asiri AM, Al-Youbi AO, Sun X. Green, low-cost synthesis of photoluminescent carbon dots by hydrothermal treatment of willow bark and their application as an effective photocatalyst for fabricating Au nanoparticles-reduced graphene oxide nanocomposites for glucose detection. Catalysis Science & Technology. 2013;3(4):1027-35.

3. Liu S, Tian J, Wang L, Zhang Y, Qin X, Luo Y et al. Hydrothermal Treatment of Grass: A Low-Cost, Green Route to Nitrogen-Doped, Carbon-Rich, Photoluminescent Polymer Nanodots as an Effective Fluorescent Sensing Platform for Label-Free Detection of Cu(II) Ions. Adv Mater. 2012;24(15):2037-41.

4. Lu W, Qin X, Liu S, Chang G, Zhang Y, Luo Y et al. Economical, Green Synthesis of Fluorescent Carbon Nanoparticles and Their Use as Probes for Sensitive and Selective Detection of Mercury(II) Ions. Anal Chem. 2012;84(12):5351-7.

5. Lu W, Qin X, Asiri AM, Al-Youbi AO, Sun X. Green synthesis of carbon nanodots as an effective fluorescent probe for sensitive and selective detection of mercury(II) ions. J Nanopart Res. 2012;15(1):1-7.

6. Liu H, Ye T, Mao C. Fluorescent Carbon Nanoparticles Derived from Candle Soot. Angew Chem, Int Ed. 2007;46(34):6473-5.

7. Feng X, Jiang Y, Zhao J, Miao M, Cao S, Fang J et al. Easy synthesis of photoluminescent N-doped carbon dots from winter melon for bio-imaging. RSC Advances. 2015;5(40):31250-4.

8. Park SY, Lee HU, Park ES, Lee SC, Lee J-W, Jeong SW et al. Photoluminescent Green Carbon Nanodots from Food-Waste-Derived Sources: Large-Scale Synthesis, Properties, and Biomedical Applications. Acs Appl Mater Interfaces. 2014;6(5):3365-70.

9. Sahu S, Behera B, Maiti TK, Mohapatra S. Simple one-step synthesis of highly luminescent carbon dots from orange juice: application as excellent bio-imaging agents. Chem Commun. 2012;48(70):8835-7.

10. Barati A, Shamsipur M, Arkan E, Hosseinzadeh L, Abdollahi H. Synthesis of biocompatible and highly photoluminescent nitrogen doped carbon dots from lime: Analytical applications and optimization using response surface methodology. Mat Sci Eng C. 2015;47:325-32.

11. Huang H, Xu Y, Tang C-J, Chen J-R, Wang A-J, Feng J-J. Facile and green synthesis of photoluminescent carbon nanoparticles for cellular imaging. New J Chem. 2014;38(2):784-9.

12. Wang L, Zhou HS. Green Synthesis of Luminescent Nitrogen-Doped Carbon Dots from Milk and Its Imaging Application. Anal Chem. 2014;86(18):8902-5.

13. Sachdev A, Gopinath P. Green synthesis of multifunctional carbon dots from coriander leaves and their potential application as antioxidants, sensors and bioimaging agents. Analyst. 2015;140(12):4260-9.

14. Qu S, Chen H, Zheng X, Cao J, Liu X. Ratiometric fluorescent nanosensor based on water soluble carbon nanodots with multiple sensing capacities. Nanoscale. 2013;5(12):5514-8.

15. Qu K, Wang J, Ren J, Qu X. Carbon Dots Prepared by Hydrothermal Treatment of Dopamine as an Effective Fluorescent Sensing Platform for the Label-Free Detection of Iron(III) Ions and Dopamine. Chemistry – A European Journal. 2013;19(22):7243-9.

16. Zhu W, Zhang J, Jiang Z, Wang W, Liu X. High-quality carbon dots: synthesis, peroxidase-like activity and their application in the detection of H_2_O_2_, Ag^+^ and Fe^3+^. RSC Advances. 2014;4(33):17387-92.

17. Zhao A, Zhao C, Li M, Ren J, Qu X. Ionic liquids as precursors for highly luminescent, surface-different nitrogen-doped carbon dots used for label-free detection of Cu^2+^/Fe^3+^ and cell imaging. Anal Chim Acta. 2014;809:128-33.

18. Huang H, Li C, Zhu S, Wang H, Chen C, Wang Z et al. Histidine-Derived Nontoxic Nitrogen-Doped Carbon Dots for Sensing and Bioimaging Applications. Langmuir. 2014;30(45):13542-8.

19. Shen C, Sun Y, Wang J, Lu Y. Facile route to highly photoluminescent carbon nanodots for ion detection, pH sensors and bioimaging. Nanoscale. 2014;6(15):9139-47.

20. Xu Q, Pu P, Zhao J, Dong C, Gao C, Chen Y et al. Preparation of highly photoluminescent sulfur-doped carbon dots for Fe(III) detection. J Mater Chem A. 2015;3(2):542-6.
